# 3,5-Diiodothyronine: A Novel Thyroid Hormone Metabolite and Potent Modulator of Energy Metabolism

**DOI:** 10.3389/fendo.2018.00427

**Published:** 2018-07-25

**Authors:** Rosalba Senese, Pieter de Lange, Giuseppe Petito, Maria Moreno, Fernando Goglia, Antonia Lanni

**Affiliations:** ^1^Department of Environmental, Biological and Pharmaceutical Sciences and Technologies, University of Campania “L. Vanvitelli” , Caserta, Italy; ^2^Department of Sciences and Technologies, University of Sannio, Benevento, Italy

**Keywords:** thyroid hormones, energy balance, mitochondria, insulin resistance (IR), obesity, fatty acids oxidation

## Abstract

Over 30 years of research has demonstrated that 3,5-diiodo-L-thyronine (3,5-T2), an endogenous metabolite of thyroid hormones, exhibits interesting metabolic activities. In rodent models, exogenously administered 3,5-T2 rapidly increases resting metabolic rate and elicits short-term beneficial hypolipidemic effects; however, very few studies have evaluated the effects of endogenous and exogenous T2 in humans. Further analyses on larger cohorts are needed to determine whether 3,5-T2 is a potent additional modulator of energy metabolism. In addition, while several lines of evidence suggest that 3,5-T2 mainly acts through Thyroid hormone receptors (THRs)- independent ways, with mitochondria as a likely cellular target, THRs-mediated actions have also been described. The detailed cellular and molecular mechanisms through which 3,5-T2 elicits a multiplicity of actions remains unknown. Here, we provide an overview of the most recent literature on 3,5-T2 bioactivity with a particular focus on short-term and long-term effects, describing data obtained through *in vivo* and *in vitro* approaches in both mammalian and non-mammalian species.

## Introduction

Thyroid hormones [3,5,3′,5′-tetraiodo-L-thyronine (T4) and 3,5,3′-triiodothyronine (T3), THs] play critical roles in differentiation, growth, and metabolism (1, 2). THs act via the nuclear thyroid hormone receptors (THRs), through different modes of action which, accordingly with Flamant (3), can be classified as: THR-dependent signaling of TH with direct binding to DNA; THR-dependent signaling of TH with indirect binding to DNA and THR-dependent signaling of TH without DNA binding; however, also THR-independent TH signaling is involved in TH mode of action (4). The different modes of action may be coupled, and several reports have recently shown that several TH metabolites act accordingly (5–8).

3, 5-diiodo-L-thyronine (3,5-T2) has emerged as a biologically active iodothyronine (9–11). Mitochondria and bioenergetic mechanisms seem to be major targets of 3,5-T2. Here, we review the most recent findings on the peripheral actions of 3,5-T2 and discuss the possible role of 3,5-T2 in the modulation of thyroid-related effects in organisms ranging from non-mammals to humans.

## The rapid effects of 3,5-T2 on energy metabolism

At 1 pM concentration, 3,5-T2 stimulates oxygen consumption more rapidly than T3 in perfused hypothyroid rat liver (12). Acute administration of T3 and 3,5-T2 to rat enhances mitochondrial activities (13, 14), with 3,5-T2 producing more rapid events (within 1 h) than T3 (after 24 h) and cycloheximide-independent (15).

The rapid increase in mitochondrial oxygen consumption by 3,5-T2 is reflected at the whole animal level (16). Injecting a single dose of 3,5-T2 (25 μg/100 g BW) to rats simultaneously administered with propylthiouracil (P) and iopanoic acid (I) (referred to as P+I, which results in severe hypothyroidism and inhibition of all the deiodinase activities) results in an increased resting metabolic rate (RMR) which is more rapid (within 6 h) than that induced by T3 (15 μg/100 g BW, effect seen within 25 h) (17). The effects following T3 injection in this study were like those reported by Tata (18), who injected the same dose. Simultaneous injection of Actinomycin D blocked the effects of T3 but not of 3,5-T2 (17), thus excluding the involvement of transcription in the effects of 3,5-T2. Indeed, it has been shown that the affinity of 3,5-T2 for human THRβ is 60-fold lower compared to T3 (19). Moreover, when injected into euthyroid rats, the effect of T3 on RMR is evident 25 h earlier than in P+I animals and is independent of Actinomycin D, suggesting that the effect of T3 injection could be due, at least in part, to the *in vivo* formation of 3,5-T2 from T3 as supported by its inhibition by P+I treatment and by the increased 3,5-T2 serum and liver levels following T3 injection into euthyroid rats (20).

The addition of nanomolar concentrations of 3,5-T2 significantly increases cytochrome oxidase (COX) activity (21) as demonstrated by specific binding of radioactive 3,5-T2 to subunit Va, and by complete reversal of its effect on respiration by a monoclonal antibody to this subunit (22, 23). The addition of 3,5-T2 to a liposome-reconstituted COX complex results in partial uncoupling which could explain its *in vivo* thermogenic effect (24). Binding sites detected by photoaffinity labeling in the rat liver cytosol (25) and by radioligand binding and displacement experiments in rat liver mitochondria (22) and cell membrane [shown by the *in vitro* activation of the Na^+^/H^+^ exchanger (26)] support the involvement of these organelles in the rapid action of 3,5-T2 (10, 26–29). The 3,5-T2 mitochondrial binding was maximal at pH 7.0 and the values for the apparent association constant and the binding capacity were 0.5 ± 0.04 × 10^8^ M^−1^ and 0.4 ± 0.04 pmol/mg mitochondrial protein respectively (21–23, 30). A top-down elasticity analysis shows that 3,5-T2 (within 1 h from injection into euthyroid rats) stimulates hepatic activity of both cytochrome c-oxidizing and -reducing components of the respiratory chain (31). 3,5-T2 also rapidly stimulates skeletal muscle mitochondrial activity and uncoupling (32, 33). 3,5-T2 rapidly increases mitochondrial Ca2+ uptake through which the iodothyronine could increase mitochondrial activity and respiration (34). More recently, the rapid effects of 3,5-T2 on intracellular Ca2+ and NO through plasma membrane and mitochondrial pathways in pituitary GH3 cells (35) further support mitochondria as a principal target of 3,5-T2 effects.

Moreover, 3,5-T2 has direct and rapid effects (within 1 h) on mitochondrial F(o)F (1)-ATP synthase activity in the liver of hypothyroid rats (36), increases mitochondrial respiration rates, increases mitochondrial uncoupling and reduces H_2_O_2_ production (37).

## The effects of long-term administration of 3,5-T2 on energy metabolism

Chronic administration of 3,5-T2 into P+I cold-exposed rats increases the energy capacity of the heart, skeletal muscle, liver, and brown adipose tissue (BAT), improving their survival in the cold (38).

Chronic administration of 3,5-T2 into P+I rats induces significant stimulation of lipid β-oxidation (39), and upregulates rat-liver mitochondrial F(o)F(1)-ATP synthase by GA-binding protein/nuclear respiratory factor-2, thus providing new insights into the 3,5-T2 role on bioenergetic mechanisms (40).

When injected into P+I rat, 3,5-T2 increases skeletal muscle lipid handling through FAT/CD36 and mitochondrial oxidation (41), activates thermogenesis, with UCP1 likely acting as the molecular determinant of this effect, and increases the sympathetic innervation and vascularization of BAT (42).

## The hypolipidemic effects of 3,5-T2

The effects of 3,5-T2 on energy metabolism has prompted research *in vitro* and *in vivo* on whether and how 3,5-T2 administration could improve adiposity and associated disorders.

## *In vitro* studies

Primary rat hepatocytes exposed to the classical oleate/palmitate (2:1 ratio) mixture have been employed as *in vitro* model of “fatty hepatocytes” to assess the effects of 3,5-T2 and T3 (doses of 10^−7^ or 10^−5^M for 24 h) on lipid metabolism (43). 3,5-T2 and T3 reduce the number and average sizes of lipid droplets, thus making stored triglycerides (TGs) more accessible to enzymes acting on the catabolism/secretion of free fatty acids. More recently, 3,5-T2 has been shown to reduce lipid excess in fatty hepatocytes by recruiting triglyceride lipase on the lipid droplet surface (44). 3,5-T2 also reduces lipid content and triggers phosphorylation of Akt in an insulin receptor-independent manner when incubated with NAFLD-like rat primary hepatocytes (45). Furthermore, 3,5-T2 enhances glucose-induced insulin secretion in both rat β-cells and human islets (46).

When exposed to an oleate/palmitate (2:1 ratio) mixture and treated with 3,5-T2 or T3 (doses of 10^−7^ or 10^−5^M for 24 h), FAO rat hepatoma cell lines, defective for functional THRs, show reduced TGs content, reduced number and size of lipid droplets and stimulated mitochondrial uncoupling (47), supporting a THR-independent TH signaling mechanisms which involve both 3,5-T2 and T3 through stimulation of mitochondrial uncoupled respiratory activity (47).

In HepG2 cells, 3,5-T2 blocks the proteolytic cleavage of SREBP-1 without affecting its expression, thus reducing fatty acid synthase expression in a way dependent on the concurrent activation of MAPK, ERK, and p38 and Akt and PKC-δ pathways (48).

## *In vivo* studies

Hypolipidemic effects have been studied *in vivo* by using several animal models (49). Simultaneous 3,5-T2 (25 μg/100 g BW) administration for 4 weeks to rats feeding a high-fat diet (HFD) prevents fatty liver and increases in body weight by increasing fatty acid oxidation rate and mitochondrial uncoupling to burn fat (50). Reductions in serum TGs and cholesterol levels (50), as well as improved insulin sensitivity (51), are also associated with 3,5-T2 administration. 3,5-T2 elicits the deacetylation of hepatic peroxisome proliferator-activated receptor gamma coactivator 1-alpha and sterol regulatory element binding protein-1c (SREBP-1c) through direct induction of silent mating type information regulation 2 homolog 1 (SIRT1) activity, resulting in increased fatty acid oxidation and decreased lipogenesis, respectively (51). Though both 3,5-T2 and T3 decrease the expression of hepatic SREBP-1c, 3,5-T2 (administered at a daily dose of 25 μg/100 g BW to high-fat diet- fed rats for 1 week), in contrast to T3 (administered at a 10-fold lower dose), does not directly induce the expression of the TRE-containing SREBP-1c lipogenic target genes [acetyl-CoA carboxylase and fatty acid synthase (52). This, at least in part, explains the effectiveness of 3,5-T2 in preventing hepatic fat accumulation and insulin resistance. Iannucci (53) showed that both 3,5-T2 and T3 exert lipolytic effects in the liver mediated by autophagy and increased fatty acid oxidation although the metabolic profiles suggested that there may be some differences in the mechanism(s) and magnitude of their metabolic effects. 3,5-T2 ameliorates muscle glucose uptake by increasing the response to insulin of Akt/PKB phosphorylation and induces structural and biochemical shifts toward glycolytic myofibers (54), thus enhancing muscle glycolytic capacity producing metabolic benefits (55–57), reminiscent of those induced by resistance exercise (58). Mitochondria adapt to the glycolytic phenotype of gastrocnemius muscle both in terms of metabolism and of dynamic with 3,5-T2 being able in reverting the HDF-associated expression pattern of proinflammatory factors (59). At the doses of 25 μg 3,5-T2/100 g BW for 4 weeks no signs of suppression of the hypothalamus-pituitary-thyroid (HPT) axis and cardiac hypertrophy are detected.

In streptozotocin-treated rats, 3,5-T2 (at the dose of 25 μg/100 g BW for 12 weeks) protects against renal damage in diabetic nephropathy through SIRT1-dependent deacetylation and inactivation of subunit p65 of NF-kB, thus inhibiting the inflammatory process related to this disease (60). Ball in rats (61), reported that 3,5-T2 is more effective in inducing hepatic malic enzyme gene expression than suppressing circulating TSH, indicating that tissue- and gene-selective effects of 3,5-T2 are not only related to differences in binding of this thyromimetic ligand to various TR isoforms but also to distinct local cellular ligand availability.

3,5-T2 administration to HFD-obese Wistar rats was also shown to reduce pre-existing hepatic fat accumulation through increased mitochondrial fatty acid oxidation coupled with less efficient utilization of substrates and reduced oxidative stress (62). A proteomic study showed that 3,5-T2 counteracts several HFD-induced changes in the protein profile, mostly in the mitochondria (63). Moreover, blue native-PAGE (BN-PAGE)/in-gel activity analysis revealed that 3,5-T2 treatment results in stimulation (vs. HFD) of respiratory complexes, thus explaining, at least in part, the anti-steatosis effect of 3,5-T2. Administration of 3,5-T2 [subcutaneously injected at doses of 25, 50, or 75 μg/100 g BW for 90 days] to chow-fed rats aged 3–6 months significantly reduces body mass and improves glucose tolerance, while heart rate and mass remain unchanged, TSH levels remain normal in rats receiving 25 μg of 3,5-T2 /100 g BW but are slightly lowered in rats that received 50 and 75 μg of 3,5-T2 /100 g BW (64). In apparent contrast, 3,5-T2 administration to Sprague Dawley rats fed a safflower-oil based HFD fails to improve NAFLD or insulin sensitivity (65). One reason for this discrepancy may be that an unsaturated fat-predominant plant oil-based diet is used (65) that could mask the hypolipidemic effects of 3,5-T2 with saturated fat-predominant animal fat-based diets (50, 51, 54, 62). Furthermore, Sprague Dawley and Wistar rats display differences in both lipoprotein metabolism and endocrine function (66).

In diet-induced obese mice, daily administration of 3,5-T2 (250 μg/100g BW for 14 or 28 days i.p.) shows beneficial effects on adiposity, serum leptin, and energy expenditure (67). The lower dose of 3,5-T2 suppress βTSH transcripts, thus suggesting a risk of interference of 3,5-T2 on the HPT axis as well as on the heart (67). Lean and diet-induced obese male mice treated for 4 weeks with a 3,5-T2 dose of 2.5 μg/g BW, show an altered expression of genes encoding hepatic xenobiotic-metabolizing enzymes involved in catabolism and inactivation of xenobiotics and TH as well as in hepatic steroid and lipid metabolism (68). Hence, the administration of this high dose of 3,5-T2 might exert adverse hepatic effects.

3,5-T2 (1.25 mg/100 g BW via daily gavage) reduces circulating total and LDL cholesterol as well as the liver level of apoB and circulating levels of both apoB48 and apoB100, but, at the same time, reduces plasma T4 levels in Western type diet fed low-density lipoprotein receptor knockout mice (69). Both 3,5-T2 and T3 administration significantly reduce nuclear HNF4α protein content, while 3,5-T2, but not T3, decreases the expression levels of the HNF4α transcriptional coactivator PGC-1α. Lower PPARα levels are found only following T3 treatment while both T3 and 3,5-T2 lower liver X receptor α nuclear content (70). 3,5-T2 (1.25 mg/100 g BW) decreases body weight and blood glucose levels through reductions in GLUT2 levels and changes in hepatic glucose output in obese mice showing to produce signs of thyrotoxicosis (71). Taken together, these studies suggest the possibility that the “thyrotoxic effects” of 3,5-T2 may be dependent upon possible differences between experiments in rats vs. mice, normal weight vs. obese, or euthyroid vs. hypothyroid animals, age, diet and temperature of exposure. It is important to note, however, that 3,5-T2 did not suppress TSH as strongly as T3 and that the cardiac readouts may represent an adaptation to increased metabolic rate, perhaps implying potential for separation of desirable effects from thyrotoxic effects.

At the current stage, no validated technique is available to accurately measure intracellular levels of 3,5-T2. Resolving this issue will bring to light to what extent 3,5-T2 is taken up in the tissues and how this relates to the effects of the exogenous administrations described above.

## The physiological and pathophysiological roles of 3,5-T2 in humans

A case report (72) involving two participants revealed that administration of 3,5-T2 to humans (1–5 μg/kg BW) rapidly (after 4–6 h) increased RMR. Chronic 3,5-T2 administration (28 days, approximately 5 μg/kg BW) increases RMR by approximately 15% and decreases body weight by approximately 4 kg in both participants. Principal clinical parameters show no significant changes and no side effects (i.e., cardiac abnormalities) are observed.

As mentioned above, reliable quantification methods to measure endogenous levels of 3,5-T2 have been lacking (73–75) and the data reported so far need independent analytical confirmation.

Recently, a mouse monoclonal antibody based on a new competitive chemiluminescence immunoassay was developed (76) to investigate the origin and action of 3,5-T2 in humans under several conditions. Data by Pietzner (77) in euthyroid human serum point toward a physiological link between 3,5-T2 (with a concentration of 0.22–0.33 nM) and glucose metabolism as well as TH homeostasis. Pietzner (78) screened the urine metabolome for associations with serum 3,5-T2 concentrations in healthy individuals, resulting in a median serum concentration of 0.24 nM. The detected metabolites are related to glucose and lipid metabolism, as well as the response to oxidative stress or drug metabolism, and are in concordance with previously published rat liver proteome analyses (63). Dietrich (79) reported elevated concentrations of 3,5-T2 (0.59 ± 0.07 nM vs. 0.39 ± 0.04) in cardiac Nonthyroidal Illness Syndrome (NTIS) suggesting that 3,5-T2 elevations in NTIS could explain why patients with low-T3 syndrome substituted with T4/T3 do not benefit from exogenous TH administration. Langouche (80), in critically ill patients reported a 30% higher serum 3,5-T2 concentration than healthy volunteers which are not independently correlated with TH.

Although recent studies in human gave some indications on the physiological and pathophysiological roles of 3,5-T2 in humans, further analyses on larger samples of euthyroid individuals are needed to obtain a more comprehensive picture of the role of 3,5-T2 in humans.

## The effects of 3,5-T2 in non-mammalian species

The effects of 3,5-T2 on metabolic efficiency is conserved across species. 3,5-T2 rapidly stimulates pyruvate-fuelled mitochondrial respiration of liver and muscle from goldfish *Carassius auratus* (81). After 12 or 24 h, 3,5-T2 rapidly decreases type 2 deiodinase (D2) activity in the liver of killifish, whilst not affecting type 1 deiodinase (D1) activity; moreover, after a 24 h exposure, 3,5-T2 (like T4 and T3) inhibits both D1 and D2 transcription (82). 3,5-T2 also regulates thermal acclimation in *Danio rerio* (83) and growth in tilapia (84). 3,5-T2 binds to and activates a specific long TRβ1 isoform that contains a nine-aminoacid insert at the beginning of the ligand-binding domain, whereas T3 can interact also with a different TRβ1 isoform that lacks this insert (19). Hernández-Puga reported that 3,5-T2 represses THRβ expression and impairs its up-regulation by cortisol possibly through a transrepression mechanism (85). Very recently, Olvera (86) reported that in tilapia cerebellum 3,5-T2 specifically regulates gene sets involved in cell signaling and transcriptional pathways, while T3 regulated pathways related to cell signaling, immune system, and lipid metabolism.

## Conclusions

Thirty years of research using mammalian and non-mammalian *in vivo* and *in vitro* models has generated substantial data on the biological effects of 3,5-T2. However, a debate is open concerning the side-effects of 3,5-T2, an issue that needs to be investigated by performing more comprehensive studies in humans and animal models to fully evaluate any potential risks.

## Author contributions

All authors listed have made a substantial, direct and intellectual contribution to the work, and approved it for publication.

### Conflict of interest statement

The authors declare that the research was conducted in the absence of any commercial or financial relationships that could be construed as a potential conflict of interest.
